# Safety and Efficacy of Direct Oral Anticoagulants for Atrial Fibrillation in Patients with Renal Impairment

**DOI:** 10.3390/pharmacy8010030

**Published:** 2020-03-04

**Authors:** Soo Min Jang, Khaled Bahjri, Huyentran Tran

**Affiliations:** 1 Department of Pharmacy Practice, Loma Linda University School of Pharmacy; Loma Linda, CA 92350, USA; Smjang@llu.edu; 2 Department of Pharmaceutical and Administrative Sciences, Loma Linda University School of Pharmacy; Loma Linda, CA 92350, USA; Kbahjri@llu.edu

**Keywords:** atrial fibrillation, chronic kidney disease, stroke prevention, thromboembolic, bleeding

## Abstract

Direct oral anticoagulants (DOACs) are gaining popularity for patients with nonvalvular atrial fibrillation (AF) for stroke prevention. Less bleeding risk with comparable stroke prevention compared to warfarin was shown. DOACs have predictable anticoagulant effects, infrequent monitoring requirements and less drug-food interactions compared to warfarin. However, safety and efficacy data of DOACs in patients with chronic kidney disease (CKD) are limited. This is a retrospective study to evaluate thromboembolic and bleeding events in patients with AF (with/without CKD) in October 2010 and July 2017. A total of 495 patients were included and only 150 patients had CKD. Our study found that patients with renal impairment on a DOAC do not have a higher incidence of bleeding events. It showed significant increase in thromboembolic events in CKD patients with dabigatran compared to CKD patients with apixaban with odds ratio of 6.58 (95%CI 1.35–32.02, p = 0.02).

## 1. Introduction

Atrial fibrillation (AF) is the most common abnormal and irregular cardiac rhythm, characterized by chaotically created electrical signals in the atria leading to loss of atrial kick and rapid, irregular ventricular contraction [1]. AF is a known risk factor for stroke, increasing the risk of stroke and thromboembolism six-fold throughout all age groups [1]. From 2012 to 2013, the direct and indirect cost of stroke was estimated to be $33.9 billion [2]. Total direct medical stroke-related costs are projected to nearly triple from $71.6 billion to $184.1 billion by 2030 [3]. AF related strokes are likely to be more severe due to the longer distance the clot needs to travel to the brain than the distance from the carotid arteries. Consequently, AF-related stroke is associated with more disability and increased mortality rate [1]. Anticoagulation therapy is crucial for stroke prophylaxis in the management of patients with AF. A measure called CHA_2_DS_2_-VASc score can help stratify risk of stroke and determine whether anticoagulants are indicated in these patients [4]. Compounded upon this increased risk, patients with concurrent chronic kidney disease (CKD) are at a higher risk for developing a thromboembolism or stroke [3,5]. As such, according to the 2019 American Heart Association/American College of Cardiology/Heart Rhythm Society (AHA/ACC/HRS) Guideline for the Management of Patients with Atrial Fibrillation, oral antithrombotic therapy is recommended for patients with nonvalvular AF and a CHA_2_DS_2_-VASc score of ≥1 in males and ≥2 in females [6].

Warfarin was the mainstay of anticoagulant therapy for thromboembolism and stroke risk reduction before the development of direct oral anticoagulants (DOACs). Compare to warfarin, DOACs have a predictable anticoagulant profile, less drug–food interactions, and less frequent monitoring requirements [7,8,9,10]. DOACs gained popularity since they showed an equivalent or better in stroke reduction with less bleeding events compared to warfarin [7,8,9,10]. However, DOACs require renal dose adjustment when warfarin does not [11,12,13,14,15]. According to the 2019 AHA/ACC/HRS Focused Update, reduced doses of dabigatran, rivaroxaban, apixaban and edoxaban are viable options in patients with moderate to severe CKD [16]. In end-stage renal disease (ESRD) patients with AF, the direct thrombin inhibitor (dabigatran) and factor Xa inhibitors (rivaroxaban or edoxaban) are not recommended due to lack of evidence suggesting that the risk outweighs the benefit [17,18,19]. The guideline still does not recommend using rivaroxaban even though it is approved to be used in ESRD patients [14,17,18,19]. Warfarin or apixaban are the preferred antithrombotic therapy in ESRD patients receiving hemodialysis (Class IIa, level B) [7]. Even though sub-analyses of major trials have shown that DOACs are safer than warfarin, patients with a creatinine clearance (CrCl) of <25 or <30 mL/min were excluded [8,9,11]. Clinicians are hesitant to use DOACs in ESRD patients (including patients receiving hemodialysis) due to scarcity of safety and efficacy data in this population [11,20]. There is insufficient evidence to provide support for that level of renal function. The purpose of this retrospective chart review is to evaluate bleeding and thromboembolic outcomes in patients (including CKD patients) with AF who managed with DOACs. We hypothesize that there will be more bleeding events in patients with moderate to severe renal impairment compared to patients with normal renal function with DOAC therapy.

## 2. Materials and Methods

### 2.1. Participants

This study is a retrospective chart review from 1 October 2010 to 1 August 2017 at the International Heart Institute at Loma Linda University Medical Center. Inclusion criteria was patients ≥18 years-old with a diagnosis of AF and treated with a DOAC (1. apixaban, 2. dabigatran, 3. rivaroxaban, and 4. edoxaban) for three months or longer. Renal function was determined by most recent lab report of serum creatinine (SCr) and calculated CrCl based on patient’s actual body weight (weight was adjusted for obese patients) with the Cockcroft–Gault equation. A total of 495 patients were included in the study, and 150 patients had calculated CrCl of <60 mL/min.

### 2.2. Data Collection

Patient demographic data, anticoagulant dose, comorbidities to calculate CHA_2_DS_2_-VASc score and HAS-BLED score, stroke events and bleeding events were collected from each patient’s electronic medical record (EMR). Our team assessed each patient’s anticoagulant dosage for the appropriateness. If a patient visited another hospital for related events, the information was still captured because they were included in our physician’s note. HAS-BLED score and CHA_2_DS_2_-VASc score were calculated based on the patient’s EMR. The predicted risk of stroke or systemic thromboembolism for all patients was assessed using the CHA_2_DS_2_-VASc scoring system (range of 0 to 9). The higher the score, the greater risk of developing stroke or systemic thromboembolism [21]. The predicted 1-year risk of major bleeding was assessed using the HAS-BLED scoring system (range of 0 to 9). The higher the score, the greater risk of major bleeding in AF patients who are on anticoagulation therapy [22].

### 2.3. Outcome

The objective of the primary outcome was to evaluate the safety of DOACs in patients with normal renal function compared to patients with varying stages of renal impairment. Patients were classified based on Kidney Disease Improving Global Outcomes criteria [23]: normal or mildly decreased renal function [estimated glomerular filtration rate (eGFR) ≥ 60 mL/min/1.73 m^2^], CKD stage 3 (eGFR = 30–59.9 mL/min/1.73 m^2^), CKD stage 4 (eGFR = 15–29.9 mL/min/1.73 m^2^), CKD stage 5 (eGFR < 15 mL/min/1.73 m^2^). Since renal dose adjustments for DOACs are recommended in patients with eGFR ≤ 50 mL/min/1.73 m^2^, patients with mildly decreased renal function (eGFR = 60–89 mL/min/1.73 m^2^) were categorized into “normal” renal function in our study. Stage 3a (45–59 mL/min/1.73 m^2^) and stage 3b (30–44 mL/min/1.73 m^2^) were merged as stage 3 in this study. A bleeding event was defined as a bleed that required discontinuation of the anticoagulant or required hospitalization. The secondary outcome was to report upon efficacy by comparing thromboembolic events in patients with normal renal function versus patients with impaired renal function. A thromboembolic event was defined as a stroke or venous thromboembolism (VTE).

### 2.4. Data Analysis

Apixaban was chosen to be our control group because it is recommended by 2019 AHA/ACC/HRS Guideline for patients even with severe renal impairment. Descriptive statistics for quantitative variables were presented using the mean with standard deviation if the values were normally distributed; median and range values were used when there were extreme outliers. Categorical variables were presented with number and percentage. Chi-square analysis was performed to report on the bleeding events and thromboembolic events amongst the CKD groups regardless of which DOAC the patient was taking. The comparison of quantitative variables between the CKD groups were performed using ANOVA when assumptions of parametric tests were met. The Kruskal–Wallis test was used to compare the medians when extreme outliers were present. Chi-square tests were also used to assess the association of the categorical variables between the CKD groups. Fischer’s exact tests were used when assumptions of Chi-square were not met. Binary logistic regression was used to assess the association of anticoagulants with bleeding events adjusted for HAS-BLED scores and thromboembolic events adjusted for CHA_2_DS_2_-Vasc scores. Significance was set at an alpha of 0.05, and statistical analysis was performed using SPSS Statistics software (version 25.0, IBM Corp. (Armonk, NY, USA)).

## 3. Results

Table 1 shows baseline demographic data of patients who were included in the study (n = 495). Patients with CKD stage 4 (n = 25) had the highest mean age of 83 ± 9 years-old and the youngest group was non-CKD patients (n = 345) mean age of 66 ± 12 years-old (p < 0.001). There were more male patients (67%) in the non-CKD patient group; more than half (>50%) were female in other groups: 54% in CKD stage 3, 60% in CKD stage 4 and 100% in CKD stage 5 (p < 0.001). Both bleeding (35.3%, p ≤ 0.001) and stroke events (16%, p = 0.007) were most frequent in CKD stage 3 group (n = 119) compared to other groups. This is an interesting finding because CKD stage 3 group had the lowest median HAS-BLED score of 2 (range 0 to 6, p < 0.001). Apixaban was most commonly used in all groups (p > 0.1).

For apixaban safety profile, 27 (17%) patients with normal kidney function, 21 (33%) patients with CKD stage 3, 6 (35%) patients with CKD stage 4 and 1 (50%) patient with ESRD had bleeding events (p = 0.017). For rivaroxaban safety profile, 20 (13%) patients with normal kidney function, 14 (33%) CKD stage 3 patients, and 1 (14%) CKD stage 4 patient showed significant differences between non-CKD and CKD stage 3 and 4 patients (p = 0.012). For dabigatran, 3 (10%) non-CKD patients and 6 (50%) CKD stage 3 patients showed significant differences in bleeding events (p = 0.013). For apixaban efficacy profile, 15 (9%) patients with normal kidney function, 9 (14%) CKD stage 3 patients, 1 (6%) CKD stage 4 patient and 2 (50%) CKD stage 5 patients developed thromboembolic events (p = 0.084). For rivaroxaban, 16 (10%) patients with normal kidney function, 5 (12%) CKD stage 3 patients, 2 (29%) CKD stage 4 patients and 1 (50%) ESRD patient (p = 0.115) reported thromboembolic events. Four (33%) patients who were taking dabigatran experienced thromboembolic events, while none of the patients (n = 1) with edoxaban had any thromboembolic events.

**Figure 1:**
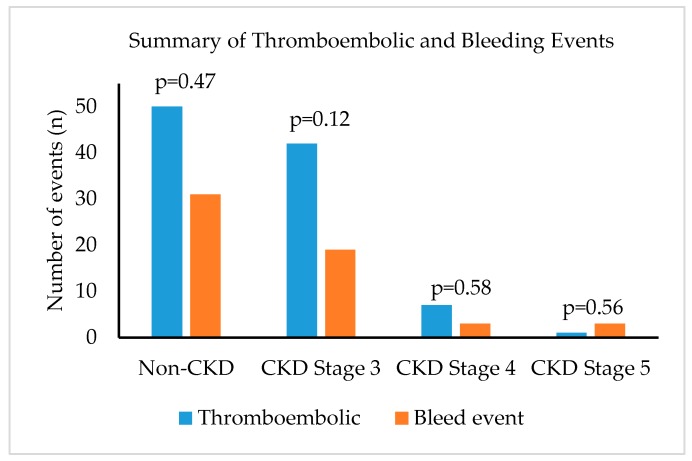
Total thromboembolic and bleeding events in different renal function. Non-CKD: >60 mL/min; CKD Stage 3: 30–59.9 mL/min; CKD Stage 4: 15–29.9 mL/min; CKD Stage 5: <15 mL/min; This figure shows summary of thromboembolic and bleeding events in all patients categorized into different renal impairment regardless of direct oral anticoagulants types.

Thromboembolic events occurred: 15% in non-CKD group, 35% in CKD stage 3 group, 28% in CKD stage 4 group, and 17% in CKD stage 5 group. Bleeding events occurred: 9% in non-CKD, 16% in CKD stage 3, 12% in CKD stage 4, and 50% in CKD stage 5. (Figure 1) When apixaban was compared to other DOACs in non-CKD patients and CKD patients (stage 3–5), there was no statistical difference in terms or bleeding events (Table 2). Table 3 shows that when apixaban was compared to dabigatran in patients with CKD (stage 3–5), adjusted odds ratio of stroke was 6.58 (95% C.I. 1.32–32.02, p = 0.02).

## 4. Discussion

To our knowledge, this is the first retrospective study that evaluated the safety and efficacy of DOACs (no warfarin) in AF patients with or without CKD. Safety and efficacy comparing warfarin as a control versus DOACs have been demonstrated in multiple clinical trials. Since the 2019 AHA/ACC/HRS focused update guideline for the management of patients with AF recommends apixaban as a preferred agent even in ESRD patients, therefore we have chosen apixaban as our control group [17]. Table 4 shows commonly used DOACs’ renal clearance, metabolism, dialyzability and renal dose adjustment recommendations. If the drug is mainly cleared by kidneys, clinicians should expect to lower the anticoagulant doses in patients with kidney diseases. Safety and efficacy data in comparison with DOACs to warfarin is also summarized in Table 4.

In our study (n = 495), DOACs did not increase the risk of bleeding events. However, there was a significant increase in thromboembolic events in CKD patients with dabigatran compared to CKD patients with apixaban. The CHA_2_DS_2_-VASc score was higher as CKD is advanced (p < 0.001). This difference may be explained because leading causes of CKD are diabetes and hypertension which contribute to the CHA_2_DS_2_-VASc scoring system. HAS-BLED scores were higher in the CKD groups compared to non-CKD group (p < 0.001). This is likely because having a renal disease is one of the criteria for a higher HAS-BLED score.

As CKD is a common comorbidity of patients with AF, there are more studies being performed and published investigating the safety of DOACs in patients with CKD [8,10,19,24]. The landmark trials, such as RE-LY, ARISTOTLE, ROCKET and ENGAGE, had subgroup analyses showing that DOACs are safe in patients with mild CKD (Table 4). Since the Cockcroft–Gault equation may overestimate patients’ renal function as CKD advances [25], RE-LY and ARISTOTLE analyzed their data using the CKD-EPI formula [26]. Similar results were shown in the RE-LY study [19], but the ARISTOTLE study showed apixaban had a favorable outcome with stroke prevention and major bleeding (p < 0.05) [27]. A study by Sarratt et al. looked at patients on dialysis requiring anticoagulation for AF [28]. A total of 120 patients were on warfarin and 60 patients were on apixaban. This study concluded that there was no statistically significant difference in thromboembolic events or bleeding events between the two group, indicating similar efficacy and safety profiles compared to warfarin. Siontis et al. recently published a retrospective cohort study (n = 25,523) to compare safety and efficacy of apixaban versus warfarin in ESRD patients with AF [29]. There was no difference in stroke or systemic embolism risks of between apixaban and warfarin, but apixaban had significantly lower risks of major bleedings.

Limitations of this study include the inherent selection bias in a retrospective chart review. The sample sizes were also unbalanced; only 150 (30%) patients had renal impairments and only 6 patients were ESRD patients. Thus, it is difficult to conclude any clinical implications in the ESRD group. Aforementioned, this study evaluates the safety and efficacy of DOACs (no warfarin) in AF patients with or without CKD. Thus, it is difficult to discuss and compare published outcome data with our results.

## 5. Conclusions

Based on this retrospective study, patients with CKD on a DOAC do not have significantly higher incidence of bleeding compared to patients with normal kidney function. However, their thromboembolic event significantly increases when patients are on dabigatran versus apixaban in patients with CKD (stage 3–5). Future randomized controlled trials comparing different types of DOAC (no warfarin) in patients with CKD are warranted.

## Figures and Tables

**Table 1:** Baseline Characteristics by levels of chronic kidney disease (CKD).

	Non-CKD (≥ 60 mL/min) n = 345	CKD Stage 3 (30–59.9 mL/min) n = 119	CKD Stage 4 (15–29.9 mL/min) n = 25	CKD Stage 5 (<15 mL/min) n = 6	p-Value
Age, mean (SD)	65.7 (12.4)	79.4 (9.3)	82.7 (9.2)	66.5 (16)	<0.001 *
Males, n (%)	231 (67)	55 (46.2)	10 (40)	-	<0.001 *
Bleeding event, n (%)	50 (14.5)	42 (35.3)	7 (28)	1 (16.7)	<0.001 *
Stroke event, n (%)	31 (9)	19 (16)	3 (12)	3 (5)	0.007 *
Antithrombotic medication					
Apixaban, n (%)	161 (46.7)	63 (52.9)	17 (68)	4 (66.7)	0.839
Rivaroxaban, n (%)	152 (44.1)	43 (36)	7 (28)	2 (33.3)	0.148
Dabigatran, n (%)	31 (9)	12 (10.1)	1 (4)	-	0.121
Edoxaban, n (%)	1 (0.3)	-	-	-	>0.999
CHA_2_DS_2_-VASc score, mean (SD)	3.2 (1.7)	4.6 (1.3)	5.4 (1.5)	5.3 (2.5)	<0.001 *
HAS-BLED score, median (range)	2 (0–9)	2 (0–6)	3 (1–4)	3 (2–5)	<0.001 *
Duration of DOAC use in months, median (range)	13 (1–73)	32 (3–65)	30 (2–60)	23.5 (3–36)	<0.001 *
History of anticoagulant use, n (%)	102 (29.6)	49 (41.2)	12 (48)	5 (83.3)	0.003 *
Concurrent antiplatelet use, n (%)	91 (26.4)	40 (33.6)	6 (24)	5 (83.3)	0.015 *

* indicates significance at an alpha of 0.05.

**Table 2:** Binary logistic regression on the effect of anticoagulants on bleeding events.

Renal Function	Anticoagulants	p-Value	Odds Ratio	95% C.I. for Odds Ratio	
				Lower	Upper
Normal kidney function	Rivaroxaban vs. Apixaban	0.464	0.79	0.42	1.49
	Dabigatran vs. Apixaban	0.413	0.59	0.17	2.09
Chronic kidney disease patients	Rivaroxaban vs. Apixaban	0.842	0.93	0.44	1.97
	Dabigatran vs. Apixaban	0.346	1.77	0.54	5.81

**Table 3:** Binary logistic regression on the effect of anticoagulants on stroke events.

Renal Function	Anticoagulants	p-Value	Odds Ratio	95% C.I. for Odds Ratio	
				Lower	Upper
Normal kidney function	Rivaroxaban vs. Apixaban	0.245	1.63	0.72	3.69
	Dabigatran vs. Apixaban	0.998	0.00	0.00	-
Chronic kidney disease patients	Rivaroxaban vs. Apixaban	0.961	0.97	0.30	3.18
	Dabigatran vs. Apixaban	0.020*	6.58	1.35	32.02

**Table 4:** Summary of renal impairment dose adjustment recommendations and primary literature for commonly used direct oral anticoagulants for atrial fibrillation [30,31,32,33,34].

DOAC	Renal CL	Hepatic Metabolism	Dialy.	Renal Impairment Dose Adjust. (mL/min)	S/SE (Compared to Warfarin)	Major Bleeding (Compared to Warfarin)
Dabigatran	80%	Metabolized by esterases	Yes	>30: 150 mg BID 15–30: 75 mg BID <15: Contraindicated	Reduced adjusted HR in CKD patients = 0.74 (95% CI 0.57 to 0.96) [35] Dabigatran 150 mg BID Overall RR [7] = 0.66 (95% CI 0.53–0.82) CrCl 30–49 mL/min = 0.85 (95% CI 0.59–1.24)	Adjusted HR in CKD patients = 1.52 (95% CI 1.27 to 1.81) [35] Dabigatran 150 mg BID Overall RR [7] = 0.93 (95% CI 0.81–1.07) CrCl 30–49 mL/min = 1.01 (95% CI 0.79–1.3)
Apixaban	25%	Mainly CYP3A4	Small	>30: 5 mg BID^^^ <30: 2.5 mg BID^+^ HD: 5 mg BID	Overall [9] = 0.79 (95% CI 0.66–0.95) CrCl 25–49 mL/min = 0.79 (95% CI 0.55–1.14)	Overall [9] = 0.69 (95% CI 0.60–0.80) CrCl 25–49 mL/min = 0.50 (95% CI 0.38–0.66)
Rivaroxaban	30%	Minimal	No	>50: 20 mg QD 15–50: 15 mg QD <15: Contraindicated	Overall HR [8] = 0.79 (95% CI 0.66–0.96) CrCl 30–49 mL/min = 0.84 (95% CI 0.75–1.23)	Overall [8] = 1.04 (95% CI 0.90–1.20) CrCl 30–49 mL/min = 0.95 (95% CI 0.72–1.26)
Edoxaban	50%	10% by carboxy-esterase 1	No	>95: FDA warning 95–50: 60 mg QD^#^ 15–49: 30 mg QD <15: Contraindicated	High-dose edoxaban Overall RR [10] = 0.79 (95% CI 0.63–0.99 CrCl 30–49 mL/min = 0.87 (95% CI 0.72–1.04) Low-dose edoxaban Overall RR [10] = 1.07 (95% CI 0.87–1.31) CrCl 30–49 mL/min = 1.22 (not reported)	High-dose edoxaban Overall RR [10] = 0.80 (95% CI 0.71–0.91) CrCl 30–49 mL/min = 0.76 (95% CI 0.58–0.98) Low-dose edoxaban Overall RR [10] = 0.47 (95% CI 0.41–0.55) CrCl 30–49 mL/min = 0.37 (not reported)

Adjust: adjustment; BID: twice daily; CL: clearance; CrCl: creatinine clearance; CYP3A4: cytochrome P450 type 3A4; Dialy: dialyzability; DOAC: direct oral anticoagulants; FDA: United States Food and Drug Administration; HR: hazard ratio; RR: relative risk; S/SE: stroke or systemic embolism; QD: once daily; ^^^ Apixaban is recommended to be dosed 2.5 mg twice daily if any ≥2 of the following criteria are met: body weight ≤60 kg, age ≥80 years-old, and serum creatinine ≥1.5 mg/dL. ^+^ Apixaban should be avoided in CrCl <25 mL/min due to lack of efficacy and safety evidence. ^#^ Edoxaban is recommended to be dosed 30 mg once daily when ≥2 of the following criteria are met: body weight ≤ 60 kg, creatinine clearance 30–50 mL/min and concomitant therapy with verapamil, dronedarone or quinidine.

## References

[1] Markides V., Schilling R.J. (2003). Atrial fibrillation: classification, pathophysiology, mechanisms and drug treatment. Heart.

[2] Nayak-Rao S., Shenoy M.P. (2017). Stroke in Patients with Chronic Kidney Disease...: How do we Approach and Manage it?. Indian J. Nephrol..

[3] Benjamin E.J., Blaha M.J., Chiuve S.E., Cushman M., Das S.R., Deo R., de Ferranti S.D., Floyd J., Fornage M., Gillespie C. (2017). Heart Disease and Stroke Statistics-2017 Update: A Report From the American Heart Association. Circulation.

[4] European Heart Rhythm A., European Association for Cardio-Thoracic S., Camm A.J., Kirchhof P., Lip G.Y., Schotten U., Savelieva I., Ernst S., Van Gelder I.C., Al-Attar N. (2010). Guidelines for the management of atrial fibrillation: the Task Force for the Management of Atrial Fibrillation of the European Society of Cardiology (ESC). Eur. Heart J..

[5] Olesen J.B., Lip G.Y., Kamper A.L., Hommel K., Kober L., Lane D.A., Lindhardsen J., Gislason G.H., Torp-Pedersen C. (2012). Stroke and bleeding in atrial fibrillation with chronic kidney disease. N. Engl. J. Med..

[6] Massicotte-Azarniouch D., Bader Eddeen A., Lazo-Langner A., Molnar A.O., Lam N.N., McCallum M.K., Bota S., Zimmerman D., Garg A.X., Harel Z. (2017). Risk of Venous Thromboembolism in Patients by Albuminuria and Estimated GFR. Am. J. Kidney Dis..

[7] Connolly S.J., Ezekowitz M.D., Yusuf S., Eikelboom J., Oldgren J., Parekh A., Pogue J., Reilly P.A., Themeles E., Varrone J. (2009). Dabigatran versus warfarin in patients with atrial fibrillation. N. Engl. J. Med..

[8] Patel M.R., Mahaffey K.W., Garg J., Pan G., Singer D.E., Hacke W., Breithardt G., Halperin J.L., Hankey G.J., Piccini J.P. (2011). Rivaroxaban versus warfarin in nonvalvular atrial fibrillation. N. Engl. J. Med..

[9] Granger C.B., Alexander J.H., McMurray J.J., Lopes R.D., Hylek E.M., Hanna M., Al-Khalidi H.R., Ansell J., Atar D., Avezum A. (2011). Apixaban versus warfarin in patients with atrial fibrillation. N. Engl. J. Med..

[10] Giugliano R.P., Ruff C.T., Braunwald E., Murphy S.A., Wiviott S.D., Halperin J.L., Waldo A.L., Ezekowitz M.D., Weitz J.I., Spinar J. (2013). Edoxaban versus warfarin in patients with atrial fibrillation. N. Engl. J. Med..

[11] January C.T., Wann L.S., Alpert J.S., Calkins H., Cigarroa J.E., Cleveland J.C., Conti J.B., Ellinor P.T., Ezekowitz M.D., Field M.E. (2014). 2014 AHA/ACC/HRS guideline for the management of patients with atrial fibrillation: executive summary: a report of the American College of Cardiology/American Heart Association Task Force on practice guidelines and the Heart Rhythm Society. Circulation.

[12] Dabigatran Etexilate Mesylate. Micromedex Solutions. Truven Health Analytics, I.A.A., MI. http://www.micromedexsolutions.com.

[13] Edoxaban. Micromedex Solutions. Truven Health Analytics, I.A.A., MI. http://www.micromedexsolutions.com..

[14] Rivaroxaban. Micromedex Solutio. Truven Health Analytics ns, I.A.A., MI. http://www.micromedexsolutions.com..

[15] Apixaban. Micromedex Solutions. Truven Health Analytics, I.A.A., MI. http://www.micromedexsolutions.com.

[16] Willett K.C., Morrill A.M. (2017). Use of direct oral anticoagulants for the prevention and treatment of thromboembolic disease in patients with reduced renal function: a short review of the clinical evidence. Ther. Clin. Risk Manag..

[17] January C.T., Wann L.S., Calkins H., Chen L.Y., Cigarroa J.E., Cleveland J.C., Ellinor P.T., Ezekowitz M.D., Field M.E., Furie K.L. (2019). 2019 AHA/ACC/HRS Focused Update of the 2014 AHA/ACC/HRS Guideline for the Management of Patients With Atrial Fibrillation: A Report of the American College of Cardiology/American Heart Association Task Force on Clinical Practice Guidelines and the Heart Rhythm Society in Collaboration With the Society of Thoracic Surgeons. Circulation.

[18] Shroff G.R. (2016). Renal Function in Patients With Atrial Fibrillation Receiving Anticoagulants: The Canaries in the Coal Mine. JAMA Cardiol..

[19] Hijazi Z., Hohnloser S.H., Oldgren J., Andersson U., Connolly S.J., Eikelboom J.W., Ezekowitz M.D., Reilly P.A., Siegbahn A., Yusuf S. (2014). Efficacy and safety of dabigatran compared with warfarin in relation to baseline renal function in patients with atrial fibrillation: a RE-LY (Randomized Evaluation of Long-term Anticoagulation Therapy) trial analysis. Circulation.

[20] Center for Drug Evaluation and Research Drug Approvals and Databases - Drug Trials Snapshot: Savaysa (edoxaban) for Prevention of Stroke in Atrial Fibrillation. U S Food and Drug Administration Home Page. https://www.fda.gov/drugs/informationondrugs/ucm428735.html.

[21] Koene R.J., Alraies M.C., Norby F.L., Soliman E.Z., Maheshwari A., Lip G.Y.H., Alonso A., Chen L.Y. (2019). Relation of the CHA2DS2-VASc Score to Risk of Thrombotic and Embolic Stroke in Community-Dwelling Individuals Without Atrial Fibrillation (From The Atherosclerosis Risk in Communities [ARIC] Study). Am. J. Cardiol..

[22] Lane D.A., Lip G.Y. (2012). Use of the CHA(2)DS(2)-VASc and HAS-BLED scores to aid decision making for thromboprophylaxis in nonvalvular atrial fibrillation. Circulation.

[23] Stevens P.E., Levin A. (2013). Kidney Disease: Improving Global Outcomes Chronic Kidney Disease Guideline Development Work Group, M. Evaluation and management of chronic kidney disease: synopsis of the kidney disease: improving global outcomes 2012 clinical practice guideline. Ann. Intern. Med..

[24] Avezum A., Lopes R.D., Schulte P.J., Lanas F., Gersh B.J., Hanna M., Pais P., Erol C., Diaz R., Bahit M.C. (2015). Apixaban in Comparison With Warfarin in Patients With Atrial Fibrillation and Valvular Heart Disease: Findings From the Apixaban for Reduction in Stroke and Other Thromboembolic Events in Atrial Fibrillation (ARISTOTLE) Trial. Circulation.

[25] Ferreira J.P., Girerd N., Pellicori P., Duarte K., Girerd S., Pfeffer M.A., McMurray J.J., Pitt B., Dickstein K., Jacobs L. (2016). Renal function estimation and Cockroft-Gault formulas for predicting cardiovascular mortality in population-based, cardiovascular risk, heart failure and post-myocardial infarction cohorts: The Heart ‘OMics’ in AGEing (HOMAGE) and the high-risk myocardial infarction database initiatives. BMC Med..

[26] Levey A.S., Stevens L.A., Schmid C.H., Zhang Y.L., Castro A.F., Feldman H.I., Kusek J.W., Eggers P., Van Lente F., Greene T. (2009). A new equation to estimate glomerular filtration rate. Ann. Intern. Med..

[27] Hohnloser S.H., Hijazi Z., Thomas L., Alexander J.H., Amerena J., Hanna M., Keltai M., Lanas F., Lopes R.D., Lopez-Sendon J. (2012). Efficacy of apixaban when compared with warfarin in relation to renal function in patients with atrial fibrillation: insights from the ARISTOTLE trial. Eur. Heart J..

[28] Sarratt S.C., Nesbit R., Moye R. (2017). Safety Outcomes of Apixaban Compared With Warfarin in Patients With End-Stage Renal Disease. Ann. Pharmacother..

[29] Siontis K.C., Zhang X., Eckard A., Bhave N., Schaubel D.E., He K., Tilea A., Stack A.G., Balkrishnan R., Yao X. (2018). Outcomes Associated With Apixaban Use in Patients With End-Stage Kidney Disease and Atrial Fibrillation in the United States. Circulation.

[30] Jain N., Reilly R.F. (2019). Clinical Pharmacology of Oral Anticoagulants in Patients with Kidney Disease. Clin. J. Am. Soc. Nephrol..

[31] Ashley J., Sood M.M. (2018). Novel oral anticoagulants in chronic kidney disease: ready for prime time?. Curr. Opin. Nephrol. Hypertens.

[32] Aursulesei V., Costache I.I. (2019). Anticoagulation in chronic kidney disease: from guidelines to clinical practice. Clin. Cardiol..

[33] Ha J.T., Badve S.V., Jun M. (2019). Recent evidence for direct oral anticoagulants in chronic kidney disease. Curr. Opin. Nephrol. Hypertens.

[34] (2019). By the American Geriatrics Society Beers Criteria Update Expert, P. American Geriatrics Society 2019 Updated AGS Beers Criteria(R) for Potentially Inappropriate Medication Use in Older Adults. J. Am. Geriatr. Soc..

[35] Lauffenburger J.C., Farley J.F., Gehi A.K., Rhoney D.H., Brookhart M.A., Fang G. (2015). Effectiveness and safety of dabigatran and warfarin in real-world US patients with non-valvular atrial fibrillation: A retrospective cohort study. J. Am. Heart Assoc..

